# Changing patterns of home visiting in general practice: an analysis of electronic medical records

**DOI:** 10.1186/1471-2296-7-58

**Published:** 2006-10-17

**Authors:** Michael J van den Berg, Mieke Cardol, Frans JM Bongers, Dinny H de Bakker

**Affiliations:** 1 NIVEL, Netherlands Institute for Health Services Research, Postal Address: PO-box 1568, 3500 BN Utrecht, The Netherlands

## Abstract

**Background:**

In most European countries and North America the number of home visits carried out by GPs has been decreasing sharply. This has been influenced by non-medical factors such as mobility and pressures on time. The objective of this study was to investigate changes in home visiting rates, looking at the level of diagnoses in1987 and in 2001.

**Methods:**

We analysed routinely collected data on diagnoses in home visits and surgery consultations from electronic medical records by general practitioners. Data were used from 246,738 contacts among 124,791 patients in 103 practices in 1987, and 77,167 contacts among 58,345 patients in 80 practices in 2001. There were 246 diagnoses used. The main outcome measure was the proportion of home visits per diagnosis in 2001.

**Results:**

Within the period studied, the proportion of home visits decreased strongly. The size of this decrease varied across diagnoses. The relation between the proportion of home visits for a diagnosis in 1987 and the same proportion in 2001 is curvilinear (*J*-shaped), indicating that the decrease is weaker at the extreme points and stronger in the middle.

**Conclusion:**

By comparison with 1987, the proportion of home visits shows a distinct decline. However, the results show that this decline is not necessarily a problem. The finding that this decline varied mainly between diagnoses for which home visits are not always urgent, shows that medical considerations still play an important role in the decision about whether or not to carry out a home visit.

## Background

Home visits are commonly seen as an important part of general practice. However, in the past decades, there has been a world-wide decrease in home visiting rates. Although there are strong variations between countries, as well as between GPs, this decrease was found in most European countries and North America [1-4].

How this decrease must be evaluated is debatable. On the one hand, this trend can be an indication of improved efficiency: GPs spend less time on less urgent home-visits, saving more time to treat patients in their practice. On the other hand, some are concerned that an essential part of general practice care might disappear and that this might lead to undesirable and dangerous situations.

Previous studies showed that home visiting rates are affected by demand, as well as supply-related factors. GPs will be more likely to visit patients who are seriously restricted in their ability to come to the practice. These restrictions can be related to age or disability but also to the complaint for which the GP is consulted. A non-medical reason for a home visit may occur if a patient has no transport.

On the supply-side, the GP's style of work has an influence. Some GPs will be more likely to address the wishes of their patients than others. The criteria for the level of discomfort that is acceptable for patients vary across GPs. Also workload related factors and the location of the practice have an influence. GPs in smaller practices make more home visits [5,6], and the proportion of elderly on the GP's list is also positively related to the number of home visits [6,7]. Furthermore, previous studies showed higher home visiting rates in rural areas than in urban areas [5,8-10].

Although the decline in home visits is generally known, very little is known about the nature of this decrease. That is to say: How does this decrease vary across different diagnoses in proportion to their urgency? The purpose of the present study was to analyse and to quantify this decrease in more detail.

The decrease in home visits indicates that GPs have sharpened their criteria for home visiting. However, GPs will still make, at least in their own point of view, responsible decisions, taking into consideration the possible discomfort or danger for the patient. This means that some complaints give more possible options than others. If a complaint appears to be very threatening, it is clear that a home visit is indicated; therefore we expect that the decrease in home visits in such cases is low. However neither do less urgent cases, on the other hand, allow the opportunity for a strong decrease. This is simply because GPs never did carry out a home visit in these cases. In other words: there is a 'bottom-effect'. The most room for making a decision about whether or not a home visit should be done, and thus for a decrease, are those complaints that are in the middle, the doubtful cases.

We expect, therefore, that the relation between the chance to get a home visit for a specific complaint and this same chance in the past is not a linear, but a *J-*shaped relation, indicating that the decrease is stronger in the middle and much smaller at the extreme points.

## Methods

Data used in this study originate from two Dutch National Surveys of General Practice (DNSGP) [11,12]. In the first DNSGP data were collected from April 1987 until March 1988 in a stratified sample of 193 general practitioners in 103 practices, who served 335.000 patients in total. In the second Dutch National Survey of General Practice data were collected during one calendar year (2001) in 104 representative general practices in the Netherlands, comprising of 195 general practitioners, who served 385.461 patients in total. The DNSGP was funded by the Dutch Ministry of Health. GPs and other care providers were asked to record every contact in an electronic medical record system. The data used in this study are the diagnosis, and the kind of contact, such as a phone call, surgery consultation, or home visit. The diagnosis was coded using the International Classification of Primary Care (ICPC). The type of contact was registered during six weeks in DNSGP2 and during three months in DNSGP1. Due to technical problems, some practices had to be excluded.

A selection of contacts was made based on two criteria. First, the diagnosis had to be registered 50 times or more in both databases. The reason for this is that under 50 percentages are determined too much by individual cases. Second, the contact had to be a face to face contact. The decision to pay a home visit is considered a two-step process. First the decision is made whether it is necessary or not to see the patient, and if not, whether a telephone consultation is an alternative. Second, whether the patient should come to the GP or the GP to the patient. Therefore, we assume that the alternative for a home visit is usually a surgery consultation. A selection of 246,738 contacts, both home visits and surgery consultations, in 1987 and 77,167 contacts in 2001, remained.

Both files were aggregated by diagnosis (ICPC-code). The variable to be aggregated was home visit (yes = 1, no = 0). In this way, for every diagnosis a proportion of home visits was computed for both years. This procedure resulted in 246 diagnoses varying from 0% to 86% home visits. Before aggregating, we weighted the data of 1987 on age and urbanization to the population of 2001. This was done to adjust for these factors, which are commonly known to influence home visits. This weighting had, however, very little influence. The un-weighted results are shown in the annex [see Additional file 1].

### Statistical analyses

The analyses were done on the level of diagnoses. Two regression analyses were conducted, using the proportion of home visits in 2001 for a specific diagnosis as the dependent variable, and the percentage of home visits for that same diagnosis in 1987 as the independent variable. In the first analysis we estimated a simple linear regression-model. Since we hypothesized that this relation is rather curvilinear, *J-*shaped, instead of linear, in the next step we added a quadratic term to the model. The whole model can now be expressed by the following equation:

Y = β_0 _+ β_1_x + β_2_x^2^

Whereby Y represents the proportion of home visits within one diagnosis in 2001 and x the proportion of home visits in 1987. Both models will be presented.

## Results

Some characteristics of the practices, patients and contacts involved in the analyses are presented in table 1. Of all face to face contacts that were included, 14.1% was a home visit in 1987 and 7.4% in 2001. Previous studies showed that of all contacts approximately 17% was a home visit in 1987 and 9% in 2001 [4]. There were a few differences between both years. The percentage of urban practices was slightly higher, the average list size was higher, which is also the case in the National population, and lastly, the average age of the patients was also slightly higher.

**Table 1:** characteristics of the practices, patients and contacts in the analyses, 1987 and 2001

	1987	2001
Registration period	13 weeks	6 weeks
		
practices	N = 103	N = 80
% single handed	50.5%	50%
% (very) urban	30.1%	42.5%
Average list size	3208	3883
Fte GP in the practice	1.4	1.6
% home visits of all face to face contacts	14.5%	7.6%
Average number of cases (contacts) in analyses	2396	964
		
Patients	N = 124,791	N = 58,345
Sex (% women)	59%	59%
Average age	39.0	42.3
Number of face to face contacts	1.98	1.32
Number of visits	0.28	0.10
		
Face to face contacts	N = 246,738	N = 77,167
% home visits	14.1%	7.4%

**Figure 1:**
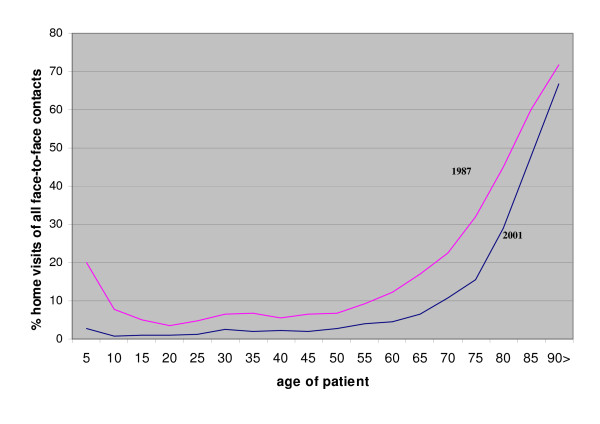
Proportion of home visits by age-cohort in 1987 and in 2001 (5-years cohorts).

Home visits are still more often carried out with the elderly people. The older the patient, the higher the chance on a home visit. This is illustrated by figure 1. The most striking difference between both years was found among the youngest patients. In 1987 significantly more home visits were carried out with children. In the youngest cohort (0 through 5 years), the percentage of home visits decreased from 20% to 3%. The proportion of home visits is also smaller among the older cohorts, especially those between the age of 55 and 75. Above that age, the difference between both years gets smaller.

Table 2 represents the results of the regression analyses. In model 1, the linear coefficient of 0.78 was found to be significant at the 0.001 level. The estimated proportion of home visits for any diagnosis is approximately 75% of the proportion in 1987. The fit of the model is quite high: 79% explained variance. In model 2 the quadratic term was added and was also found to be significant at the .001 level. This leads to 4% additional explained variance. The proportion for 2001 can now be expressed as: 0.01+ 0.36 times the proportion in 1987, plus 0.66 times the square of this proportion. These results confirm the hypothesized *J-*shaped relationship.

**Table 2:** Relation between proportion home visits in 2001 (dependent) and the proportion of home visits in 1987 for a diagnosis (n = 246 diagnoses) (regression analyses)

	Model 1a	Model 1b
Constant	-0.03	0.01
Proportion 1987	0.78**	0.36**
(proportion 1987)^2^		0.66**
		
R^2 ^(0 through 1)	0.79	0.83

* *p < .001

**Figure 2:**
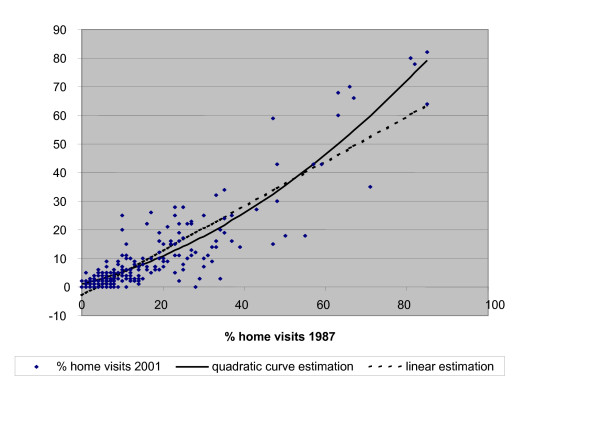
Scatter plot with quadratic regression curve of the proportion of home visits per diagnosis in 2001 in relation to the percentage of home visits in 1987 (n = 246 diagnoses).

To get a better insight, both regression lines are displayed in figure 2. When for a diagnosis only 20% of the contacts resulted in a home visit in 1987, in 2001 the estimated proportion is 11%. 40% in 1987 becomes 26% in 2001, 50% becomes 36%. At the level of 80% in 1987 there is still a decrease of 7% but when we reach 90% or more, there is hardly any decrease. Theoretically, at the proportion of 96%, the estimated proportion in 2001 exceeds the proportion in 1987. However, such high proportions do not really exist in the file.

Obviously, some diagnoses are closer to their predicted value than others. Although the model fits very well, there are some diagnoses that show relatively high differences between both years. Table 3 shows the top-5 of diagnoses with the strongest decreases in the proportion of home visits. These are: fever; acute myocardial infarction; osteoporosis; concussion; and tonsillitis, angina, and scarlatina. In only a few diagnoses there is a contrast to the overall trend, a higher proportion of home visits in 2001 than in 1987. This was the case for 'generalized pain' (A01) and acute stress-reaction (P02).

**Table 3:** Five strongest decreases: (1987>2001)

	ICPC	Diagnosis	Proportion 1987	Proportion 2001	Difference*	Prevalence^1^
1	A03	Fever	0.55 (0.49–0.61)	0.18 (0.13–0.23)	-0.37	6.7
2	K75	Acute myocardial infarction	0.71 (0.63–0.79)	0.35(0.25–0.45)	-0.36	3.3
3	L95	Osteoporosis	0.50 (0.44–0.56)	0.18 (0.10–0.26)	-0.32	4.2
4	N79	Concussion	0.47 (0.42–0.53)	0.15 (0.05–0.25)	-0.32	1.8
5	R72	Tonsillitus/angina/scarlatina	0.34 (0.26–0.42)	0.03 (0.0–0.07)	-0.31	1.7

* all differences are significant; p < 0.005

^1 ^prevalence per 1000 patients, per year in Dutch general practice in 2001 [13]

## Conclusion and discussion

By comparison with 1987, the proportion of home visits shows a distinct decline. We expected that this decrease was not equal for all kind of diagnoses, but relatively stronger for the complaints 'in the middle', with median proportions, and smaller at the extreme points. Our findings lend support for this hypothesis.

One plausible explanation for this finding is that every home visit is the outcome of the weighting of discomfort and, or danger, for the patient on one hand and the discomfort, for example in the amount of time spent, for the GP on the other hand. Better transport facilities for patients and an increase of the workload experienced over a period of time might have loaded the latter factor. It is obvious that in very severe cases these non-medical factors are of less importance. The more threatening a complaint, the less room the GP has for making medical and other decisions. This finding suggests that the decrease in home visits is not necessarily a problem. There seems to be no reason to assume that GPs take unacceptable risks since medical factors are still taken into consideration. In urgent cases, most GPs still visit their patients.

An explanation for some large decreases is that medical knowledge and commonly accepted ideas about specific complaints have changed. In the list of strongest decreases, fever, streptococcal infections and concussion can be traced back to altered views in medical management. Fever in itself is no reason for a visit, in the case of concussion, advice can often be given without seeing the patient. The reason that patients with a myocardial infarction have fewer visits, is likely to be related to the more active therapeutic approach adopted since 1987. Many of them undergo a PTCA within the first days after their infarction and within a week they leave the hospital. In 1987 the treatment was more often conservative, the patients stayed longer in the hospital and were discharged with restrictions on exercise. It is not plausible that the decrease involves the first emergency calls when a patient experiences chest pain. However, the design of our study does not differentiate between several types of visits. The place of osteoporosis in the top-five decreases is difficult to interpret within the limits of this study.

Although the results showed that the decrease in home visiting rates become smaller when the complaints become more urgent, there is a decrease in the overwhelming majority of the complaints. The finding that GPs do more visits when the patients report acute stress reactions or psychological symptoms, is surprising in the light of the declining number of visits. An explanation might be that in case of serious psychological symptoms, it is easier for the GP to visit these patients than receiving them in their practice. So, in such cases it is both in the interest of the GP and the patients to carry out a home visit. Moreover, when an emotionally stressed and possibly confused patients calls, it is often difficult to make an estimation of the urgency of the complaint [14].

What does this information mean for the GP? First, the results show that some complaints provide more room for manoeuvre in the choice of whether or not to carry out a home visit. Furthermore, in the discussion over whether or not the decrease in home visiting is problematic, this information supports the claim that GPs who reduce their number of home visits do not necessarily make irresponsible decisions.

The purpose of this study was to describe the relationship between GPs and their patients in very broad outlines in order to get an insight into the overall pattern of the decrease in home visits on the level of complaints and diagnoses. Therefore we used aggregated data and created abstract research entities. The characteristics of patients, GPs, practices and their context have been shown to play an important role in home visiting but were beyond the scope of this study. However, more insight into the nature of the decrease in home visits can be an important point of departure for more explanatory studies.

## Competing interests

The author(s) declare that they have no competing interests.

## Authors' contributions

MJB was involved in the original idea, design, analysis and interpretation of the data, wrote the major part of the manuscript.

MC was involved in the original idea and design and contributed to the writing and revision of the manuscript

FJMB contributed to the writing and critical revision of the manuscript and to the interpretation of the data

DHB Was involved in the data collection and the design of the DNSGP and contributed to the critical revision of the manuscript and the interpretation of the data.

All authors read and approved the final manuscript.

## Pre-publication history

The pre-publication history for this paper can be accessed here:



## Supplementary Material

Additional file 1 ANNEX un-weighted results. The data provided represent the relation between proportion home visits in 2001 (dependent) and the proportion of home visits in 1987 (as in table 2) and the five strongest decreases, these data are un-weighted. Click here for file
