# Supplementary material for: Changing patterns of home visiting in general practice: an analysis of electronic medical records
Source: BMC Fam Pract. 2006 Oct 17;7:58. doi: 10.1186/1471-2296-7-58 (PMC1624836; doi:10.1186/1471-2296-7-58)
Supplement: Additional file 1 — ANNEX un-weighted results. The data provided represent the relation between proportion home visits in 2001 (dependent) and the proportion of home visits in 1987 (as in table 2) and the five strongest decreases, these data are un-weighted. [file 1471-2296-7-58-S1.doc]

# ANNEX un-weighted results

**Table 2a: Relation between proportion home visits in 2001 (dependent) and the proportion of home visits in 1987 for a diagnosis (n=246 diagnoses)**

(regression analyses, un-weighted data)

|  | Model 1 | Model 2 |
| --- | --- | --- |
| Constant | -0.03 | 0.01 |
| Proportion 1987 | 0.78** | 0.32** |
| (proportion 1987)^2^ |  | 0.73** |
|  |  |  |
| R^2^ (0 thru 1) | 0.79 | 0.83 |

* *p<.001

Table 3a: Five strongest decreases: (1987>2001) un-weighted data

|  | ICPC | Diagnosis | Proportion 1987 | Proportion 2001 | Difference* | Prevalence ^1^ |
| --- | --- | --- | --- | --- | --- | --- |
| 1 | A03 | Fever | 0.53 (0.47-0.59) | 0.18 (0.13-0.23) | -0.36 | 6.7 |
| 2 | R72 | Tonsillitus/  angina/  scarlatina | 0.36 (0.27-0.45) | 0.03 (0.0-0.07) | -0.33 | 1.7 |
| 3 | K75 | Acute myocardial infarction | 0.68(0.59-0.77) | 0.35( 0.25-0.45) | -0.33 | 3.3 |
| 4 | L95 | Osteoporosis | 0.48 (0.42-0.54) | 0.18 (0.10-0.26) | -0.30 | 4.2 |
| 5 | N79 | Concussion | 0.44 (0.38-0.49) | 0.15 (0.05-0.25) | -0.29 | 1.8 |

* all differences are significant ( p<0.005)

^1^ prevalence per 1000 patients, per year in Dutch general practice in 2001 [13]
